# Parent/caregiver perspectives of meaningful improvement in functional domains for people with CDKL5 deficiency disorder: a mixed-methods study

**DOI:** 10.1007/s11136-025-04048-0

**Published:** 2025-09-09

**Authors:** Jessica Keeley, Zoe Skoda, Karen Utley, Eric D. Marsh, Gabrielle A. Conecker, JayEtta Hecker, Natasha N. Ludwig, Helen Leonard, Jacinta Saldaris, Peter Jacoby, Sharon Pincus, Tim A. Benke, Scott T. Demarest, Jenny Downs

**Affiliations:** 1 The Kids Research Institute Australia, The University of Western Australia, P.O. Box 855, West Perth, WA 6872 Australia; 2 School of Psychology, Murdoch University, Perth, Australia; 3 International Foundation of CDKL5 Research, Wadsworth, USA; 4 Division of Neurology, Children’s Hospital of Philadelphia, School of Medicine, University of Pennsylvania, Philadelphia, PA USA; 5 International SCN8A Alliance, a Project of Decoding Developmental Epilepsies, Washington, DC USA; 6 Kennedy Krieger Institute, Center for Neuropsychological and Psychological Assessment & Johns Hopkins School of Medicine, Department of Psychiatry & Behavioral Sciences, Baltimore, MD USA; 7 Adult & Child Center for Outcomes Research & Delivery Science/The NavLab, University of Colorado Anschutz Medical Campus, Aurora, CO USA; 8 Children’s Hospital Colorado, Paediatrics and Neurology, University of Colorado School of Medicine, Aurora, CO USA; 9 Department of Pediatrics and Neurology, School of Medicine, University of Colorado Precision Medicine Institute, Children’s Hospital Colorado, Aurora, CO USA; 10 Curtin School of Allied Health, Curtin University, Perth, Australia

**Keywords:** CDKL5 deficiency disorder, Developmental and epileptic encephalopathy, Priority domains, Meaningful improvement, Mixed methods

## Abstract

**Purpose:**

CDKL5 deficiency disorder (CDD) is a rare developmental and epileptic encephalopathy. Greater understanding of the smallest meaningful improvements for individuals with CDD in clinical trials and practice is needed for a person-centred approach to treatment efficacy. This study explored how parent/caregivers of people with CDD understood meaningful improvements and described change for priority functional domains including communication, gross motor, fine motor, feeding.

**Methods:**

This study included an in-person workshop and a convergent mixed-methods online survey. Parent/caregivers (*n* = 19) attending the 6th Family Educational and Awareness Conference for CDD participated in discussion groups for the workshop component. The survey (*n* = 80) collected descriptive data and open-ended responses. Qualitative data were stratified by ability levels and analysed using a conventional content analysis.

**Results:**

Definitions of meaningful improvement varied in terms of desired speed and magnitude of change and included health and skill stability. Parent/caregivers described meaningful increases in developmental skills that were specific to each domain and level of ability. Some concepts were common across ability levels within domains (e.g., increased independence across all gross motor levels) and others were consistent across domains (e.g., improved utensil use in fine motor and feeding domains).

**Conclusion:**

Meaningful improvement means different things to different people with some factors consistent regardless of ability level suggesting important underlying concepts for measurement requiring future investigation. These findings can contribute to the development of clinical treatments and trials that focus on factors that are important to people with CDD and their families.

## Plain English summary

CDKL5 deficiency disorder (CDD) is a condition that effects an individual’s development and almost always involves seizures from a very young age. Scientific advancements mean that new treatments are coming. We need to be able to accurately measure change in individuals to know if these new treatments are effective. However, it is not enough to just be able to measure change, we also need to know what change would be meaningful to the individual and their families (i.e., meaningful change). This study explored how parent/caregivers of a child with CDD understand meaningful change for communication, gross motor (body movements), fine motor (hand movements) and feeding, and examined the similarities and differences for different ability levels. We found that meaningful change meant different things to different people, where desired change could have been large, small, long-term, ongoing, or fast. Parent/caregivers also wanted stability (i.e., no changes) which is new information and adds to how meaningful change can be defined. Some meaningful changes were common across domains (e.g., improved engagement with others) and others were unique to specific domains (e.g., develop ability to sit). Understanding what developmental changes are meaningful helps to plan what to measure in clinical trials and how to understand the changes that are achieved.

## Introduction

Advances in genetic diagnostic technologies have identified over 800 genetic aetiologies for developmental and epileptic encephalopathy (DEE) conditions and the number is increasing [1]. DEEs are rare and usually associated with severe impacts on health and development. CDKL5 deficiency disorder (CDD) is a DEE condition that has been estimated to affect approximately 1:40,000 and 1:60,000 live births [2]. CDD is characterised by early-onset treatment-resistant seizures and profound impairments across multiple developmental domains including gross motor, fine motor, communication, and cognition, as well as health domains including gastrointestinal, visual, and sleep [3].

To date, evaluations of new symptomatic treatments in DEE conditions focused on measurement of seizures [4], with minimal focus on documentation of the broader set of impairments including high priority domains of daily living such as communication, mobility, and feeding [5, 6]. Simplifying the suite of outcomes measured in complex epilepsy conditions to control seizures neglects caregiver-defined priorities for CDD [7, 8], creating a disconnect between trial objectives and meaningful improvement for families. Precision medicine trials aim to modify the disease and generate substantive impacts on multiple symptoms where a broader suite of measures of functional abilities and comorbidities, including epilepsy, would indicate improvement.

Accordingly, a suite of fit-for-purpose clinical outcome assessments (COA) are needed to best document clinical progress in DEEs. Evidence of the reliability and validity of COAs in DEE populations is accumulating [e.g., 9, 10]. Clarity on meaningful change and improvements desired by parent/caregivers is a critical accompaniment to traditional reliability and validity metrics and is usually considered as the minimal important difference (MID) or the smallest difference that is clinically important to patients [11]. More recently, the Food and Drug Administration has included focus on individual level change rather than between group difference described in MID values [12]. It is not known how parent/caregivers with a child with a DEE conceptualise meaningful change and improvement as a concept, and there are limited data on individual level improvement that would be meaningful by domain [13]. Addressing these gaps are crucial for ensuring that patient-centred outcomes and caregiver-expressed priorities inform the capacity of evaluations in clinical trials and practice to identify what matters to patients and their caregivers.

This mixed-methods study explored caregiver perspectives on meaningful improvement and variations in expressed across ability levels in the domains of communication, gross and fine motor skills, and eating abilities in people with CDD. This study comprised two parts, each with their own research question(s).

1. Part 1 Workshop: How do parent/caregivers of people with CDD conceptualise meaningful improvement?
2. Part 2 Survey: (a) How do parent/caregivers describe meaningful improvement for different levels of ability in functional domains; and (b) What are the common and unique meaningful improvement factors across ability levels and domains?

## Methods

**Fig. 1:**
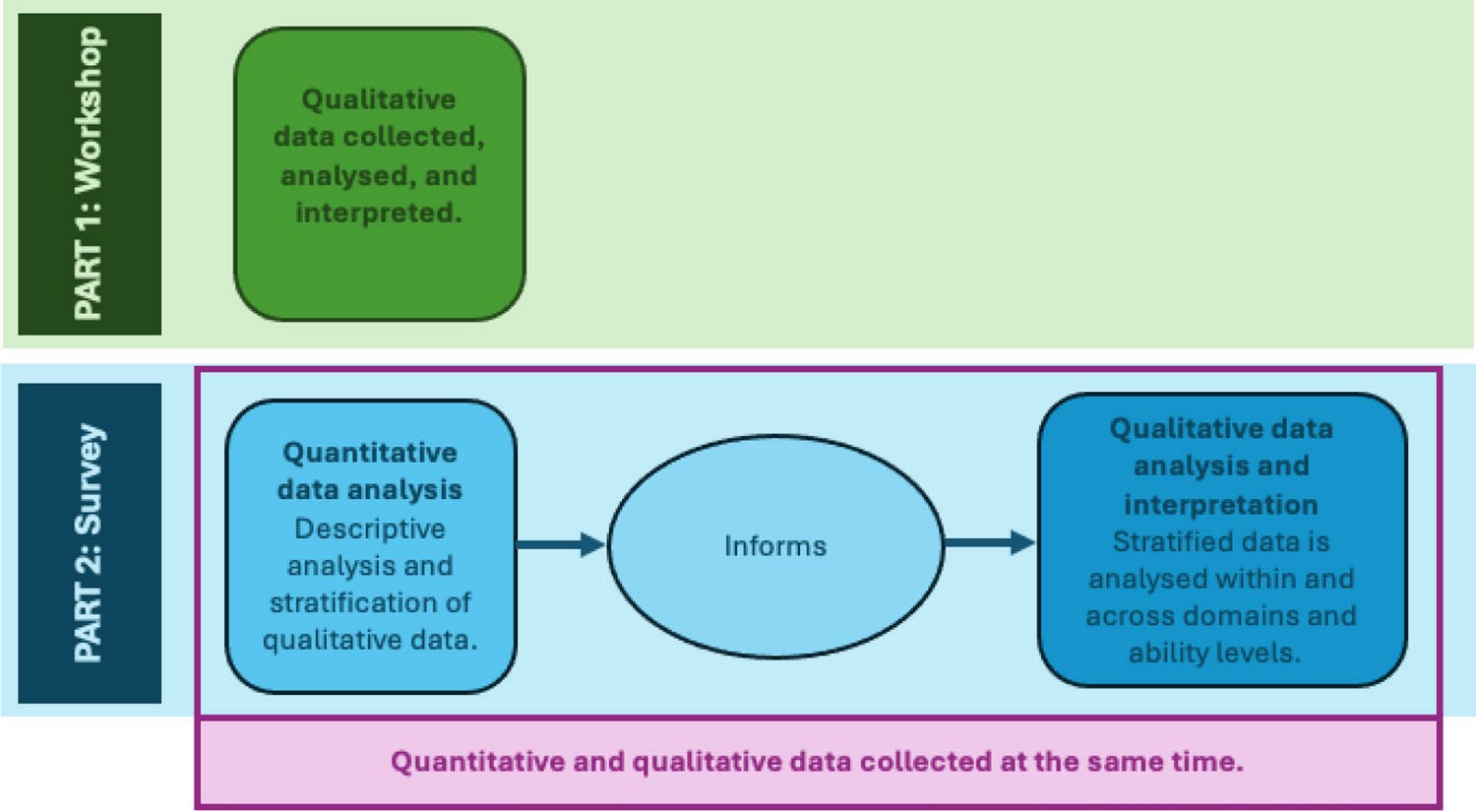
Flow of data collection, analysis, and interpretation

This study involved an in-person workshop with parent/caregivers of people with CDD and a convergent mixed-methods study design where quantitative and qualitative data were collected together [14]. The workshop was conducted at the 6th Family Educational and Awareness Conference for CDD in Cleveland USA in 2024 and an international online survey was administered. This approach allowed for the exploration of the concept of meaningful improvement in CDD and insights into specific conceptualisations of meaningful improvement by domain and for different ability levels within domains. The workshop and survey were analysed and interpreted separately. See Fig. 1 for the flow of data collection and analysis of findings.

This study was approved by the University of Western Australia Human Research Ethics Committee (2019/RA/4/20/6198). Informed consent was provided in writing prior to engaging in the workshop and electronic informed consent was provided for the online survey before commencing. Parent/caregivers of people with CDD were consulted in the design of this study.

### Part 1: Conference workshop

The workshop convened at the 6th Family Educational and Awareness Conference in Cleveland Ohio on the 15th June 2024 which is organised by the International Foundation for CDKL5 Research and is held approximately biennially. This conference aims to support the information needs of parent/caregivers of people with CDD by addressing caregiving challenges and presenting recent clinical care and research findings. A family conference setting was used to capitalise on capacity for face-to-face discussions with parent/caregivers.

#### Participants

Parent/caregivers who attended the conference were invited to participate. Nineteen parent/caregivers (15 mothers, 4 fathers) of 19 people with CDD took part. The session was advertised in the conference program and all parent/caregivers who attended were informed that taking part in the workshop involved participating in the study. The inclusion criterion was that participants were a caregiver of an individual with CDD. No one refused to participate or withdrew from the study.

#### Procedure

**Fig. 2:**
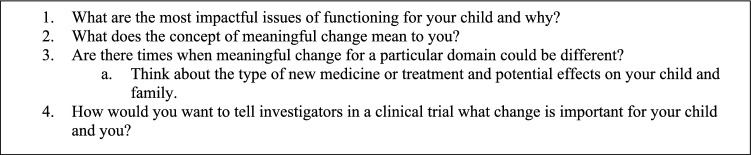
Workshop questions for small group discussions

The workshop was led by JD and lasted approximately 75 min. This included a short presentation on the concept of meaningful improvement and some information about the parallel survey, followed by a 40-min facilitated small group discussion. Each group included a facilitator who was a researcher and/or clinician with expertise and experience in CDD. Groups discussed each question shown in Fig. 2 and the facilitator made brief notes on paper, laptops, or Post-it notes which reflected discussed concepts. These were collected for analysis.

### Part 2: Online survey

#### Participants

The survey was advertised in the International Foundation for CDKL5 Research newsletters and at the Family Educational and Awareness Conference. Parent/caregivers of a person with CDD who wished to participate were provided with a link to the online survey. Of the 135 surveys, 50 were excluded from the analysis of this study because they were incomplete (participants could not return to the survey if they could not complete it in one sitting) or described children who were younger than 12 months (*n* = 5). Data were collected from 80 parent/caregivers, 70 (87.5%) of whom had a confirmed genetic test for CDD. The remaining participants identified their child as having CDD (including a date of diagnosis) but genetic confirmation was not available to the researchers. Functional ability and seizure data indicated that these children experienced severe impairments (e.g., all were currently experiencing seizures and none could walk independently). Eight parent/caregivers participated in both the workshop and the survey.

#### Procedure

The survey was devised by the research team and members of the International Foundation for CDKL5 conference committee. It was hosted on REDCap, comprised two sections, and took approximately 20 min to complete. Section one asked for demographic and descriptive and information including questions about epilepsy, cortical vision impairment (CVI), and difficulties with behavioural regulation.

Section two addressed four core domains of functioning including communication, gross motor, fine motor, and feeding. These domains have been identified as priorities by parent/caregivers of people with CDD and similar conditions [7] and were sensible categories within which meaningful improvement can be explored. Parent/caregivers provided descriptive information about their child’s current abilities according to the following categories.

1. ***Communication:*** A modified version of the Communication Function Classification System [CFCS] people [15] was used to describe three levels of communication ability including (1) effectively communicates back and forth with known and new people, (2) communicates back and forth with known people but less effective with new people, and (3) seldom or never communicates effectively even with familiar people.
2. ***Gross motor:*** Modified from previous work [16], responses for sitting and walking were combined to describe gross motor skills as (1) can sit and walk independently, (2) can sit independently but not walk, and (3) cannot sit or walk independently.
3. ***Fine motor:*** Levels of hand function was organised into (1) can pick up small objects, (2) can pick up large objects, and (3) unable to pick up or grasp objects [10].
4. ***Feeding safety and independence:*** A modified version of the Eating and Drinking Ability Classification System [EDACS; 15] classified abilities to eat safely into (1) eats and drinks safely (2) eats and drinks with some difficulty, (3) very difficult to eat or drink safely. As previously [17], eating independently was classified as (1) feeding self at mealtimes including using utensils, (2) able to finger feed but needs help with utensils, and (3) needs to be fed.

#### Open-ended questions

Parent/caregivers were then asked to describe treatment goals and the smallest improvement resulting from treatment that would be meaningful for their child’s function and quality of life.

### Data management and analysis for Part 1 and Part 2

Data from Part 1 were analysed first. Workshop data from facilitator notes were compiled, organised, and analysed in Excel. A conventional content analysis was conducted following the guidelines outlined by Hsieh and Shannon [18]. This descriptive approach explored concepts by identifying categories of information without applying predetermined or theory driven concepts [18]. ZS organised the data before JK completed the coding by first applying codes to the data and then organising the codes into relevant and informative groups. Multiple codes were applied where different meanings were observed. The codes were thoroughly discussed within the research team to ensure that their application was accurate and representative.

For the survey, descriptive quantitative survey data were analysed in Statistics and Data Science (Stata version 18.0, standard edition) by ZS and JD. Quantitative data describing levels of ability for each domain were used to stratify participant responses. The qualitative data were analysed within these levels of ability in Excel by ZS one domain at a time using a conventional content analysis. Common and unique concepts were identified and organised through a process of coding. This process included applying meaningful codes that represented concepts in the data to segments of data. Codes were derived from the data rather than predetermined (i.e., inductive) and multiple codes were applied where different meanings were evident. JK also coded all the communication domain data and 30% of each of the other domains. Some minor differences were deliberated and 7% of the reviewed codes were altered. Codes were reviewed within and across the levels of ability and unique and common concepts were identified.

#### Trustworthiness

The trustworthiness of the qualitative analysis was enhanced in multiple ways, in addition to researcher triangulation (confirmability) described above. Parents/caregivers were consulted at two points during the study to ensure that the research was acceptable to the community and represented their needs. First, an online meeting was held with three parents/caregivers of people with CDD during the planning stage of the study. The draft outline of the study was shared, discussed, and changes were made in response to feedback. Second, three parent/caregivers of people with CDD (one also attended the first meeting) discussed preliminary findings during an online meeting. Members commented that our interpretation of meaningful improvement was consistent with their own understanding and experiences, enhancing the credibility of the findings. Further, an audit trail was maintained throughout to increase dependability (e.g., meeting notes including group decisions and different document tabs in an excel file for each stage of analysis to reflect on and confirm decisions).

## Results

### Part 1

**Fig. 3:**
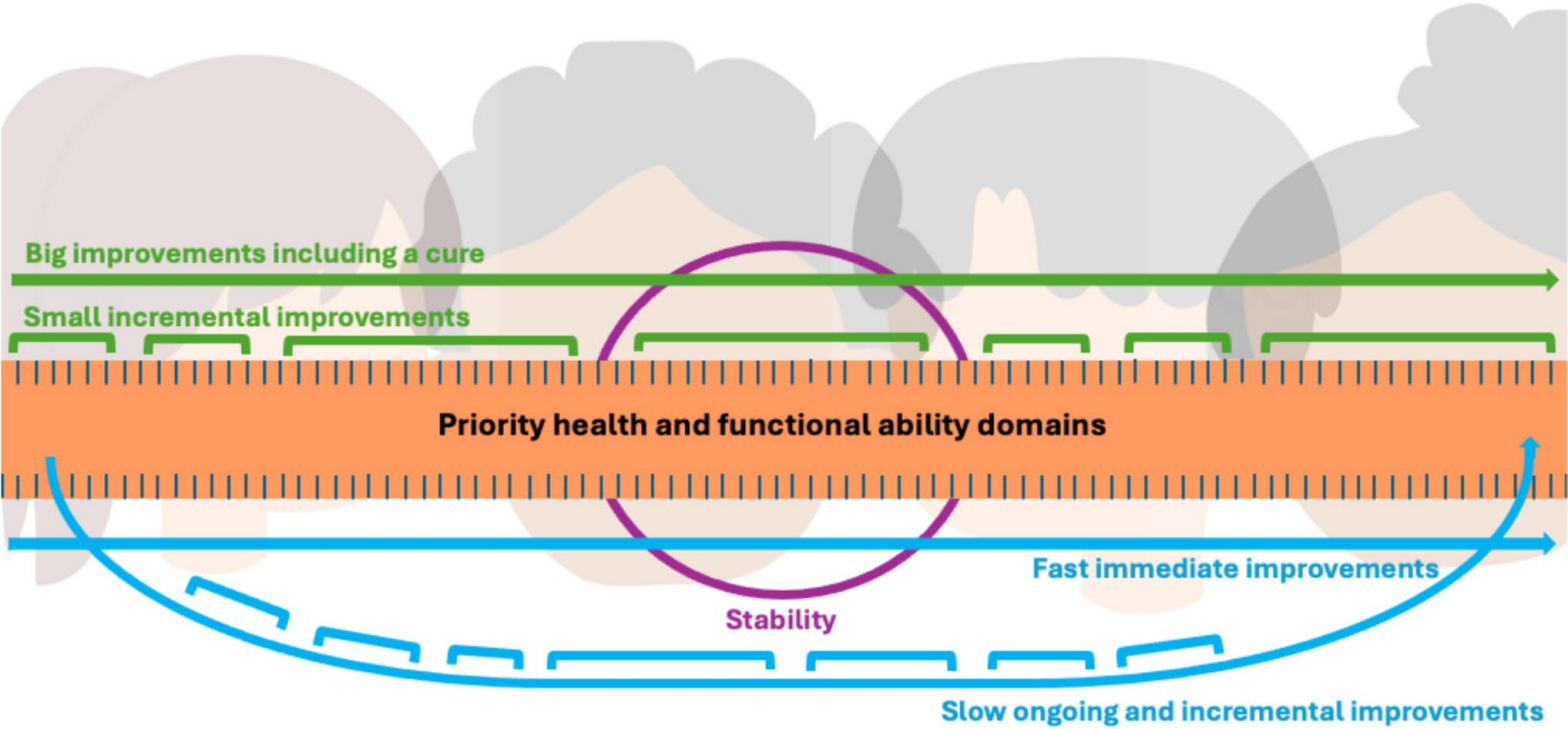
Part 1 findings: parent/caregiver conceptualization of meaningful improvement in CDD. *Note* Meaningful improvement was defined and measured (ruler) by parent/caregivers differently (different faces). Magnitude of improvement is represented by the green lines. Some parent/caregivers wanted big improvements including a cure (green line below the ruler). While some wanted any magnitude of improvement (green lines above the ruler), others wanted symptom stability (purple circle). Speed of improvement is represented by the blue lines. Some parent/caregivers wanted immediate and fast improvements (straight blue arrow) while others were satisfied with slow and ongoing improvement (curved blue arrow with smaller blue leaps)

Ninety codes were identified in the workshop data. Understandings of meaningful improvement varied across the sample in terms of desired magnitude and imminency (Fig. 3). Desired magnitude of improvement ranged from small or any improvement (e.g., “Any reduction in seizure activity—no amount would be too small”) to large improvements or a cure (e.g., “Complete seizure control/epilepsy cured”). Long-term and ongoing improvements (e.g., “Meaningful change is an incremental change that would move them toward that goal”) were desired by some. Parent/caregivers also identified stability as a meaningful improvement described as consistency and predictability of symptoms, behaviour, and functional abilities. For example, one note read “Stability = meaningful change” and another “can plan” indicating that stability would allow them to plan their daily lives and manage their child’s health with greater regularity and less variability.

Discussions between parent/caregivers and facilitators indicated multiple needs and priorities. Communication, seizure management, and challenging behaviours were often mentioned, as well as desired improvements in social participation, sleep, feeding, gross motor, and fine motor skills. The improvements were described in terms of how they would impact the individual (e.g., improved quality of life, greater independence) and the family (e.g., improved mental wellbeing, better balance for child and family needs).

### Part 2

The final sample comprised caregivers of 68 (85%) females and 12 (15%) males with CDD, aged between 1 and 59 years (41% 1 < 6 years). Participants included biological mothers (*n* = 69, 86%), biological fathers (*n* = 10, 13%), and one carer (1%) of a child with CDD, and most lived in North America (70%). Almost all had current problems with seizures (94%), more than half had a diagnosis of CVI (62%), 25% walked independently (including those who could walk without difficulty, with some difficulty, and short distances only), and few used spoken words (12%; Table 1). Most participants who responded to the descriptive items provided qualitative meaningful improvement data (*n* = 76 communication, *n* = 78 gross motor, *n* = 75 fine motor). Qualitative data on feeding safety and independence were not requested from those who exclusively used a feeding tube. Feeding safety included 46 responses and feeding independence included 52 responses.

**Table 1:** Description of the individuals with CDD (*N* = 80)

Variable	Level	n (%)
Age group (years)	1–5	33 (41)
	6–12	19 (24)
	13–20	16 (20)
	21+	12 (15)
Sex	Male	12 (15)
	Female	68 (85)
Location of residence	North America	56 (70)
	Europe	19 (24)
	Other^a^	5 (6)
Diagnosed with CVI	No	16 (20)
	Yes	50 (63)
	Features of CVI but no diagnosis	14 (17)
Current problems with epilepsy/seizures	No	5 (6)
	Yes	75 (94)
Behavioural regulation challenges	No	49 (61)
	Yes	31 (39)
Communication with others^b^	Effectively communicates back and forth with known and new people	4 (5)
	Communicates back and forth with known but less effective with new people	23 (29)
	Seldom or never communicates effectively even with familiar people	54 (66)
Gross motor	Can sit and walk independently	16 (20)
	Can sit but not walk independently	48 (60)
	Cannot sit or walk independently	16 (20)
Fine motor	Can pick up small objects	21 (26)
	Can pick up large but not small objects	19 (24)
	Unable to grasp objects	39 (49)
	No response provided	1 (1)
Feeding tube	No	47 (59)
	Yes	20 (25)
	Yes, but partly eats by mouth	13 (16)
Eating safety^c^	Eats and drinks safely	25 (42)
	Eats and drinks with some difficulties	29 (48)
	Very difficult to eat or drink safely	6 (10)
Eating independently^c^	Feeds self and manages mealtime activities but may need some guidance/help	7 (12)
	Can finger feed but needs help to eat with utensils	12 (20)
	Needs to be fed	41 (68)

^a^Other comprises Oceania, Asia, South America

^b^Modified Communication Function Classification System [CFCS; 15]

^c^Those who were exclusively tube feed (*n* = 20) were not presented the safety and independence questions. Percentages provided are for available responses (*n* = 60)

A total of 107 unique codes were applied to the data. Unique and common codes for each level of ability within each domain are summarised below (Table 2). Unique factors were mostly domain specific and indicated how improvements would be demonstrated (e.g., new skill development or advancement). Generally, common factors indicated the perceived outcome of the developmental improvement (e.g., improved independence). For example, in the gross motor domain, a unique factor was to improve walking ability (i.e., a domain related skill) and a common factor was to improve engagement with others and the environment (i.e., an outcome of improved skills). Parent/caregivers described improvements in the child’s current functioning across all levels of ability. For children classified in the less complex skill groups, parent/caregivers sometimes wanted to regain previously held skills rather than attaining new skills (Table 2).

**Table 2:**
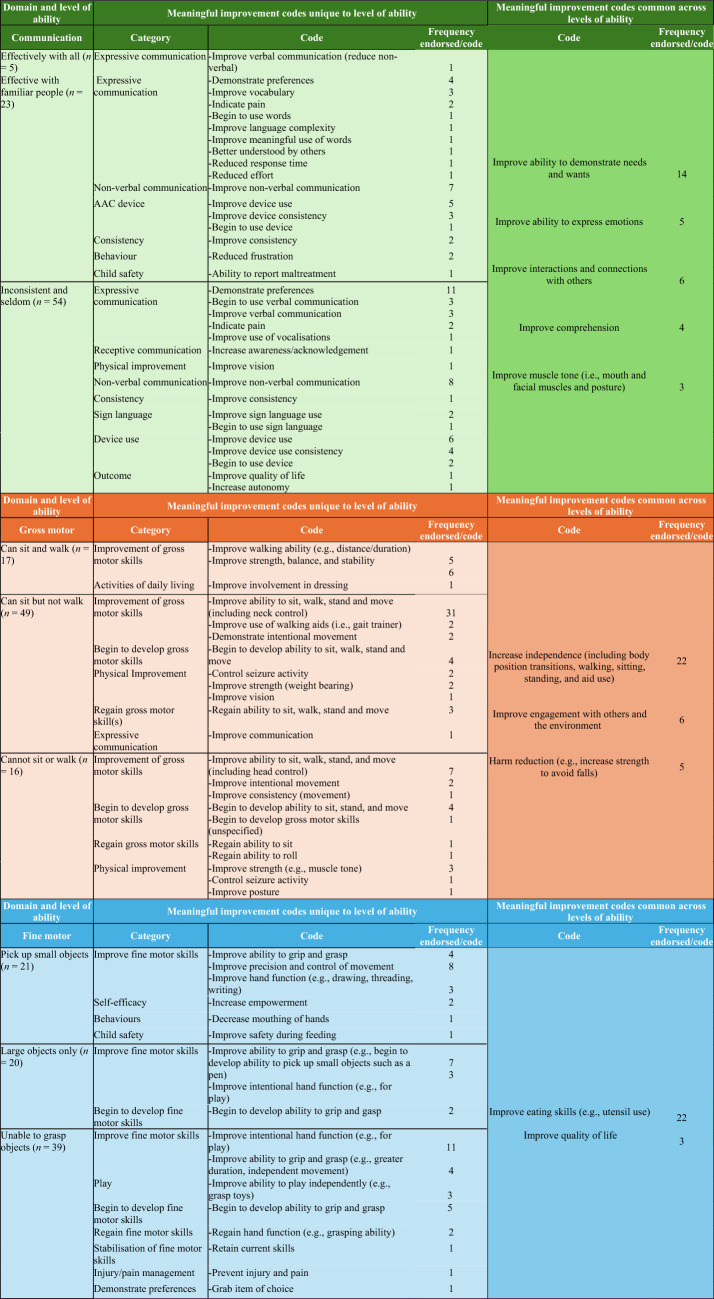

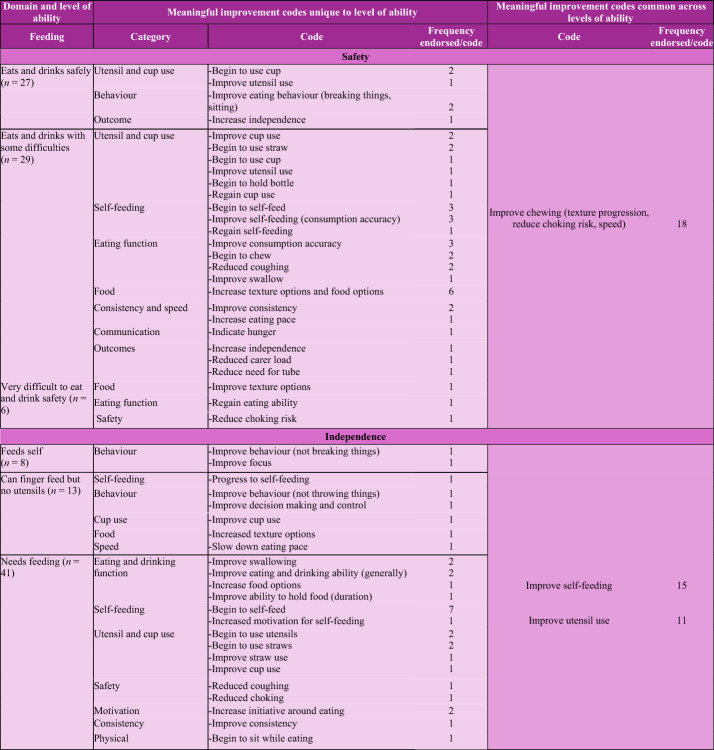
Common and unique meaningful improvement codes for each functional domain, classified by levels of functional ability and categories of improvement codes

Responses were assigned multiple codes where different concepts were identified. Those who exclusively tube feed (*n* = 20) were not presented the safety or independence questions. Outcomes describe the intended result of a meaningful improvement for the individual or family

#### Communication

Caregivers consistently wanted to better understand their child’s needs, wants (*n* = 14), and emotions (*n* = 5,), for their child to make better connections with others (*n* = 6), and for improved comprehension (*n* = 4), and muscle tone (*n* = 3) across all levels of ability. Some commonly desired improvements in the group who communicated effectively only with known people and those who inconsistently and seldomly communicated with others included demonstrating preferences (*n* = 15) and improved use of non-verbal communication (*n* = 15).

#### Gross motor domain

Across all levels of ability, parent/caregivers desired increased independence (for movement or daily living tasks, *n* = 22), engagement (with others and the environment, *n* = 6), and safety for their child (*n* = 5). Parent/caregivers described improvements specific to the category of their child’s current abilities. For example, for those who could sit but not walk, developing the ability to walk was desired. Improved ability to sit, walk stand and move (including head control) was the most common code and was found in those who could sit but not walk (*n* = 31) and those who could neither sit nor walk (*n* = 7).

#### Fine motor domain

For fine motor, parent/caregivers consistently wanted improved eating skills (*n* = 22) and better quality of life (*n* = 3) across all ability levels. Improved ability to grip and grasp was identified in both the pick-up small objects (*n* = 4) and pick up large objects only (*n* = 7) groups. The most common unique code was improving intentional hand function (e.g., for play, *n* = 11) identified in the unable to grasp objects group. Improvements and new skills were desired by the parent/caregivers with lower hand function abilities, and the development of more complex skills (e.g., drawing) was desired for those with more advanced skills. For some in the unable to grasp objects, regaining or retaining hand function was desired.

#### Feeding domain

Feeding was organised into two parts: safety and independence. Codes addressing safety and independence appeared across both sub-domains. For example, reduced choking risk was identified in the safety and independence sub-domains as were progress to (*n* = 4) and improving self-feeding skills (*n* = 4). Compared to the other domains, the feeding domains had fewer codes that were consistent across all the levels of ability, most likely due to the small numbers in some groups.

##### Safety

Improved ability to chew was described as a meaningful improvement for feeding safely across all levels of ability (*n* = 18). Increased ability to eat different food textures was the most common unique code (*n* = 7) reported for individuals who could eat and drink but with some difficulty. One parent/caregiver reported that that feeding was time consuming and that an increase in independent feeding would result in more time and energy for the caregiver (i.e., reduced carer load).

##### Independence

Meaningful improvement for independent feeding was identified as improving ability to self-feed (*n* = 15) and use eating utensils (*n* = 11), across all levels of ability. Unique codes were found for each of the groups, although comparisons were limited due to the small sample size.

### Common factors across domains

Some codes and concepts were consistent across different domains. Ability to be consistent was described in the communication (general and device use, *n* = 16), eating safely and independently (*n* = 3), and gross motor (*n* = 1) domains. Desire for greater independence was identified in the gross motor (*n* = 22), fine motor (*n* = 7), eating safely (*n* = 2), and eating independence domains. Demonstrating preferences (*n* = 16) and better quality of life (*n* = 4) were described for the communication and fine motor domains. These factors may represent universally important meaningful improvements for parent/caregivers in their children with CDD.

## Discussion

This mixed-methods study explored how parent/caregivers of individuals with CDD understood meaningful improvement and how their child’s level of functional ability impacted this view. The findings suggest that parent/caregivers perceived multiple definitions of meaningful improvement, including clinical stability which has not been described before. Across each level of ability, parent/caregivers viewed gaining additional skills as meaningful. Some meaningful improvements were common across domains (e.g., improved engagement with others) and others were unique to specific domains (e.g., develop ability to sit in the gross motor domain). Parent/caregivers wanted their child to develop, improve, regain, or avoid losing skills across all domains and ability levels.

The concept of stability as a form of meaningful improvement was consistent across the workshop and survey data and appears to be novel in the meaningful improvement literature. Traditionally, meaningful change is conceptualised as improvement in a health outcome as perceived by the target population [19, 20] and is mostly considered in relation to gain or loss of skills [21, 22]. Concordant with disease complexity and refractory epilepsy in CDD [3, 23] some parent/caregivers described wanting consistency of symptoms and behaviours as a form of meaningful improvement. This suggests that variability of daily health and developmental status is a significant challenge for families [24], often involving rapid deteriorations, intensive medical care in the home and the hospital. Stability (i.e., avoiding sudden exacerbations of seizures, respiratory infections or other symptoms, or deterioration in skills) represented a meaningful improvement. Future research should seek to understand how achieving stability can be measured and considered when evaluating therapeutics, which traditionally aim to reduce symptoms.

Some meaningful improvement codes were reported across domains suggesting that some outcomes or consequences of meaningful improvement are broadly functional and not specific to the skills in the domain. For example, independence was identified in all but one domain and many of the fine motor codes centred around feeding and improved utensil use like those in the feeding domains. Other examples included demonstrating preferences and improving social connections identified in the communication and gross motor domains. Ability to communicate preferences contributes to child agency and quality of life in people with CDD [25]. Similarly, connecting with others demonstrated by enjoyment with smiles and laughter in social interactions can contribute to quality of life [25]. Understanding the impacts of meaningful improvement on quality of life is important as both parents [26] and children [4, 27] with rare diseases and DEEs are at risk of lower quality of life than those affected by other conditions. The linkages of meaningful improvement concepts across domains should be considered in future measurement development.

Accurate measurement of meaningful improvement is necessary to determine the efficacy of treatments from a person-centred perspective. Findings from the current study are consistent with recent research describing meaningful change for functional ability domains reported for SCN2A DEE [13] and in multiple DEE conditions [6, 28] where there are severe impacts on health and development. When asked in the current survey about meaningful improvement, parent/caregivers commented on both skills (e.g., ability to walk) and more distal outcomes (e.g., increased independence), suggesting that measurement of meaningful improvement needs items both for specific skills and broader outcomes for a more complete understanding [28]. Generated from concept elicitation, the current data form a platform for studies that would aim to identify meaningful improvements in skills and scores in relevant outcome measures for CDD, as has been conducted for Dravet syndrome [21] and Duchenne muscular dystrophy [22]. Evidence is accumulating to inform the development of a single meaningful improvement measurement tool for future therapy trials targeted at CDD and other DEE conditions.

### Strengths and limitations

Understanding what meaningful improvement is to parent/caregivers is needed for interpretation of the impacts of upcoming gene therapy trials in DEEs. The mixed methods approach used in our study was a strength as it allowed for exploration of meaningful improvement within and between different levels of functional abilities. The two qualitative components complemented and reinforced each other as a form of triangulation. This study also benefitted from the collaboration and guidance of the International Foundation for CDKL5 Research.

This study had several limitations. First, this study focused on functional domains and excluded domains including seizures, vision, and behaviour which are important topics for future research. Second, Part 1 was part of a conference workshop where capacity for administration was limited, resulting in the use of less precise and exhaustive data collection methods (e.g., Post-it notes). Focus groups and individual concept elicitation interviews could allow for recording of richer discussions that could be transcribed and analysed in greater depth and with more accuracy. Third, the conference may have excluded the perspectives of parents with children with more complex medical needs (e.g., frailty, intensive respiratory care needs) and families with less financial means who have limited capacity to travel. However, the survey data was not limited by these restrictions. Fourth, these findings primarily represent the perspectives of mothers and not fathers which may be different [29] and should be explored in future research. Finally, for feeding safely, a survey error meant that qualitative data were not requested from those who identified that their child’s feeding was safe, efficient, and independent.

## Conclusion

Meaningful improvement was variable among parent/caregivers of people with CDD. Some meaningful improvements within domains were consistent across ability levels while others were unique, and other codes were common across different domains. These findings can inform the setting and interpreting of clinical trial endpoints and potentially, the development of a broader pan-domain outcome measure that measures meaningful improvement in alignment with the needs of individuals with CDD and their families, contributing to a more patient-centred approach to clinical trials and research.

## Data Availability

These data are not available to the public to ensure that participant information remains confidential and private.
